# Bioactivity of Wild Hop Extracts against the Granary Weevil, *Sitophilus granarius* (L.)

**DOI:** 10.3390/insects12060564

**Published:** 2021-06-19

**Authors:** Gianluca Paventi, Giuseppe Rotundo, Marco Pistillo, Ilaria D’Isita, Giacinto Salvatore Germinara

**Affiliations:** 1 Department of Medicine and Health Sciences “V. Tiberio”, University of Molise, via de Sanctis, 86100 Campobasso, Italy; 2 Department of Agricultural, Environmental and Food Sciences, University of Molise, via de Sanctis, 86100 Campobasso, Italy; 3 Department of Agricultural Sciences, Food, Natural Resources and Engineering, University of Foggia, via Napoli 25, 71100 Foggia, Italy; marco.pistillo@unifg.it (M.P.); ilaria.disita@unifg.it (I.D.)

**Keywords:** botanical insecticides, pest control, plant extracts, *Humulus lupulus*

## Abstract

**Simple Summary:**

One of the outstanding problems in pest control is the extensive use of synthetic compounds characterized by concerns such as risks to non-target organisms, slow degradation, and development of resistance. For these reasons, the interest in more ecofriendly pesticides, such as botanicals, is progressively increased in the last two decades. In this regard, having recently found that essential oil obtained by wild hop has biological activity against *Sitophilus granarius*, here we checked whether and how three different crude extracts obtained by the same hop ecotype also presented toxicity (contact, ingestion, inhalation) and/or repellent activity against the same insect, which is one of the most damaging pests of stored products. Results reposted here clearly show that, in addition to the essential oil, hop crude extracts (methanol, acetone, and *n*-hexane) preserve interesting activities against pests. Moreover, since they can be easily obtained and produce high yields, hop crude extracts could represent a valid tool for *S. granarius* control.

**Abstract:**

The use of bioinsecticides, rather than synthetic compounds, appears a goal to be pursued in pest control, especially for species such as *Sitophilus granarius* (L.) which attack stored products. Since *Humulus lupulus* (L.) is a remarkable source of bioactive compounds, this study investigated the bioactivity of hop flower extracts against *S. granarius* adults by evaluating toxic (contact, inhalation, and ingestion), repellent, antifeedant, and nutritional effects as well as their anticholinesterase activity and olfactory sensitivity. Hop extracts were obtained by soaking dried and ground hop cones in solvents of decreasing polarity: methanol, acetone, and *n*-hexane. Dried crude extracts were resuspended in each solvent, and used in topical application, ingestion, and fumigation toxicity assays, as well as in contact and short-range repellency tests, in vitro anticholinesterase activity evaluation, and electroantennographic tests. No inhalation toxicity for the extracts was found. On the contrary, all extracts showed adult contact toxicity 24 h after treatment (LD_50/_LD_90_ 16.17/33.20, 25.77/42.64, and 31.07/49.48 µg/adult for acetone, *n*-hexane, and methanol extracts, respectively); negligible variations for these values at 48 h were found. The anticholinesterase activity shown by all extracts suggested that the inhibition of this enzyme was one of the mechanisms of action. Interestingly, flour disk bioassays revealed a significant ingestion toxicity for the acetone extract and a lower toxicity for the other two extracts. Moreover, all extracts affected insect nutritional parameters, at the highest dose checked. Filter paper and two-choice pitfall bioassays showed repellent activity and a strong reduction of insect orientation to a highly attractive food odor source, with minor differences among extracts, respectively. Finally, the presence of volatile compounds in the different extracts that are perceived by insect antennae was confirmed by electroantennography. All these findings strongly suggest a possible use of hop cone extracts against *S. granarius*, thus further confirming this plant as an interesting species for pest control.

## 1. Introduction

Botanical pesticides represent a valuable alternative to synthetic chemicals since their use significantly reduces the risk to non-target organisms due to their rapid degradation in the environment; moreover, by providing novel and multiple modes of action, the probability of pest resistance development is reduced as well. Thus, the Integrated Pest Management (IPM) approach [1,2,3] strongly encourages the search for novel active botanicals. Accordingly, the number of papers describing plant extracts active against different pests is progressively increasing, as shown by a simple search using the keyword couple “plant extract” and “pest” (Scopus database, https://www.scopus.com, accessed date 19 February 2021) which returned 20, 185, 679, and 1304 results for the decades 1981–1990, 1991–2000, 2001–2010, and 2011–2020, respectively. In this regard, extracts and/or essential oils from several plants proved to exert insecticidal activity against vegetables and stored product pests and have been proposed for practical application [4,5,6,7]. Among the plethora of plant species investigated [8,9,10,11,12], hop, *Humulus lupulus* (L.), is receiving increasing interest for possible re-utilization after commercial use (e.g., beer production) and for its bioactivity against bacteria, yeast, fungi, and insects [13,14,15,16]. Besides spent hop, wild hop also showed remarkable activity against pests, such as Colorado potato beetles (*Leptinotarsa decemlineata* Say) (Coleoptera: Chrysomelidae) [17,18,19], as well as noxious insects and other invasive species [14,20]. Recently, wild hop essential oil (EO) and its main constituents (α-humulene, β-myrcene, and β-caryophyllene) proved to exert interesting properties against the granary weevil, *Sitophilus granarius* (L.) (Coleoptera, Curculionidae): A high contact and a lower inhalation toxicities, as well as a good repellent activity, were found against adult insects [21]. On the other hand, the limited yield of hop EO extraction may limit its use for pest control purposes. Moreover, the insecticidal activities of EOs and crude extracts of the same plant may significantly vary due to the different nature of extracted compounds [22]; in addition, home-made plant crude extracts could offer a low-cost acceptable alternative for farmers [5]. To provide a wider scenario on the hop biological activity in insects, the insecticidal, electrophysiological, and behavioral activities of *n*-hexane, methanol, and acetone crude extracts from wild hops of Central Italy were investigated against the granary weevil adults.

## 2. Materials and Methods

### 2.1. Chemicals

Solvents (methanol, n-hexane, and acetone) and all other chemicals were obtained from Sigma–Aldrich (Milan, Italy) and were at the purest grade available.

### 2.2. Plant Material

Aerial parts of hop, *Humulus lupulus* (L.) Cannabaceae, were collected in September 2019 during the flowering stage in Bojano (Molise region, Italy) at 482 m a.s.l. The area (N 41°47′840″ E 14°49′428″) had an average annual rainfall of 700 mm, and mean annual temperature of 14–15 °C. The soil where hop plants were harvested has neutral pH (7.25), a sandy texture (fine sand 54%, coarse sand 23%), a low organic carbon content (10.7 g/Kg), and a low C/N ratio (5.9), as measured in [23]. It is also a strongly calcareous soil (CaCO_3_ 37.26%), with a very low content of available phosphorus (P_2_O_5_ 5.14 mg/kg) for plants. Voucher specimen n. 20,348 was deposited in the herbarium of the University of Molise.

### 2.3. Insect Rearing

*Sitophilus granarius* were reared for several generations (2 years) at the Department of Agriculture, Environment and Food of the University of Molise. Insects were maintained on wheat grains (*Triticum aestivum* L., cv Bologna) in glass cylindrical containers (Ø 15 × 15 cm) closed by metallic net (1 mm) put in the dark in a climatic chamber set at 25 ± 2 °C and 60 ± 5% r.h. Adult beetles, 2–4 weeks old, were used only once for the experiments.

### 2.4. Plant Extracts

Hop cones were oven-dried at 36 ± 2 °C for 72 h and ground, and aliquots of powder (50 g) were extracted for 24 h at room temperature using solvents with different polarity: methanol, acetone, or *n*-hexane (250 mL). Then, each crude extract was filtered (Whatman No. 113, Cytiva, Marlborough, MA, USA), dried under vacuum in a rotary evaporator (Laborota 4000, Heidolph, Schwabach, Germany), and stored at −20 °C until further use. The residues obtained were 126.9 ± 20.6, 121.1 ± 3.3, and 96.6 ± 11.6 g/kg dry weight (mean values ± SE) for methanol, acetone, and *n*-hexane extracts, respectively.

### 2.5. Contact Toxicity

The contact toxicity of hop extracts to granary weevil adults was determined by topical application [8,24]. Plant extract samples were prepared by dissolving the residues of *n*-hexane, methanol, and acetone extracts in *n*-hexane, acetone:methanol (1:1), and acetone, respectively. For each sample, two-fold serial dilutions (150.00–4.69 μg/μL) were prepared and for each dilution an aliquot (0.5 µL) was applied on the pronotum of *S. granarius* adults in thanatosis using a Hamilton’s syringe (700 series, MicroliterTM Hamilton Company, Reno, NV, USA). Each dilution was assayed on 40 unsexed adults of *S. granarius* divided in 8 replicates and an equal number of individuals was treated with the respective solvent as a control. For each replicate, insects were confined in a Petri dish within a metal rings (Ø 4.0 × 2.5 cm), covered with metallic net (mesh 1 mm) to prevent insects escape, with 5 wheat kernels, and maintained in the dark under controlled conditions (26 ± 2 °C and 60 ± 5% r.h.). Insect mortality was recorded after 24 and 48 h. The percentage mortalities were transformed to arcsine square-root values for one-way analysis of variance (ANOVA). Treatment means were compared and separated by Tukey’s HSD test. The lethal dose 50 (LD_50_) and 90 (LD_90_) values, the confidence upper and lower limits, regression equations, and chi-square (χ^2^) values were calculated using probit analysis [25]. Statistical analyses were performed with SPSS (Statistical Package for the Social Sciences) v.23 for Windows (SPSS Inc., Chicago, IL, USA).

### 2.6. Inhalation Toxicity

The inhalation toxicity was assessed by using a fumigation chamber made up of a plastic container (135 mL) in which a perforated septum separated a lower chamber from an upper one. The lower chamber was assigned to contain increasing doses (2.5–150 mg) of each hop extract residue whilst the upper chamber contained adult weevils (*n* = 20) and intact kernels (*n* = 20). For each sample three replicates were set up. Dead insects were counted, after incubation in the dark at 26 ± 2 °C and 60 ± 5% r.h. for 24 or 48 h.

### 2.7. Ingestion Toxicity, Antifeedant, and Nutritional Activity

Effects of hop extracts on the feeding activity and nutrition of granary weevil adults were evaluated by the flour disk bioassay [8,26]. Wheat flour (10 g) was uniformly suspended in distilled water (50 mL) by magnetic stirring. To obtain flour disks, aliquots (200 μL) of suspension were dropped onto a plastic Petri dish and left overnight at 26 ± 2 °C and 60 ± 5% r.h. to dry. Plant extract samples and their dilutions were prepared by dissolving the residues as described in Section 2.5. Disks were treated with sample solutions (5 μL) corresponding to different concentrations (46.87, 93.75, 187.50, 375.00, 750.00 μg/disk) or the solvent alone as a control. Disks were held at room temperature for 2 h for solvent evaporation. In a pre-weighed glass vial (Ø 2.5 × 4.0 cm) 2 flour disks were introduced and the weight measured; later, 10 group-weighed weevil adults were added and each vial was re-weighed and maintained in the dark at 26 ± 2 °C, 60 ± 5% r.h. for 5 days. At the end of the test, for each glass vial, insects were removed, the number of dead insects recorded, and the weight of both the 2 flour disk residues and live insects were separately measured. As a control, glass vials containing treated flour disks but without insects were prepared to determine any decrease in weights due to evaporation of solvent and sample. For each sample concentration, as well as for the control, 5 replicates were set up. The following nutritional indices [4,19,20] for each replicate were calculated:

Relative Growth Rate (RGR) = (A − B)/B × day^−1^ (1)

Relative Consumption Rate (RCR) = D/B × day^−1^ (2)

Efficiency Conversion of Ingested food (ECI) = (RGR/RCR) × 100(3)

Feeding Deterrence Index (FDI) (%) = [(C − T)/C] × 100(4)

where A = mean weight (mg) of live insects on fifth day; B = initial mean weight (mg) of insects; C = consumption of control disks; D = biomass ingested (mg)/no. of living insects on the fifth day; and T = consumption of treated disks.

Data were submitted to ANOVA followed by Tukey’s HSD test for mean comparisons.

### 2.8. AChE Assay

Anticholinesterase activity of hop extracts was investigated [8,21] by detecting AChE activity photometrically (λ = 412 nm, 25 °C) by means of a Jasco V-570 spectrophotometer (Tokyo, Japan). Briefly, about 0.01 EU of enzyme (EC 3.1.1.7, from *Electrophorus electricus*, Sigma–Aldrich, Milan, Italy) were incubated in phosphate buffer (0.1 M, pH 8.00) plus 5,5′dithiobis(2-nitrobenzoic) acid (DTNB, 0.2 mM) either in the absence or in the presence of different aliquots of hop extracts. For *n*-hexane extract, the addition of Tween-20 (0.4%, *v*/*v*) allowed its re-suspension in phosphate buffer. Reaction was started by the addition of saturating concentration (2.5 mM) of acetylthiocholine iodide and the rate of absorbance change was obtained as tangent to the initial part of the progress curve. IC_50_ values were calculated by means of Grafit 4.0 (Erithacus Software Ltd., East Grinstead, UK). Results were expressed as % of the control (reaction rate measured in the absence of plant extract). Data were submitted to ANOVA followed by Tukey’s HSD test for mean comparisons.

### 2.9. Two-Choice Behavioural Bioassays

The capability of different hop extracts to disrupt granary weevil orientation to odors of wheat grains was evaluated in a two-choice pitfall bioassay [27]. The test arena was a steel container (Ø 32 × 7 cm height) with two diametrically opposed holes (Ø 3 cm) located 3 cm from the side wall. A filter paper disc (Ø 0.7 cm) was suspended at the center of each hole by a cotton wire taped to the lower surface of the arena. Glass flasks (500 mL), assigned to collect the responding insects, were positioned under each hole. The inside necks of the collection flasks were coated with mineral oil to prevent insects from returning to the arena. Thirty unsexed insects, left for at least 4 h without food, were placed under an inverted Petri dish (Ø 3 cm × 1.2 cm high) at the center of the arena and allowed 30 min to acclimatize prior to release. During the assay, the arena was covered with a steel lid to prevent insects from escaping. In each experiment, insects were given a choice between the odors emitted by wheat grains (200 g; 14.5% moisture content) left in a collection flask alone or plus a set dose (10 µL) of an extract solution adsorbed onto the overlying filter paper disc and, as a control, the respective solvent (10 µL) adsorbed onto the opposed paper disc. In each set of experiments, five doses (0.094, 0.188, 0.375, 0.750, 1.500 mg) of each hop extracts were assessed. Tests lasted 3 h and were carried out in the dark at 26 ± 2 °C and 60 ± 5% r.h. Each bioassay was replicated 4 times. In each experiment, a response index (RI) was calculated by using:

RI = [(T − C)/Tot] × 100(5)

where T is the number responding to the treatment, C is the number responding to the control, and Tot is the total number of insects released [28]. For each test stimulus, the significance of the mean RIs was evaluated by comparing the mean number of insects in the treatment and control using a Student’s *t*-test for paired comparisons. The mean numbers of insects found in the treatment and in the control and the mean RIs at different doses of hop extracts alone and in the presence of wheat grain odors were subjected to ANOVA and ranked according to Tukey’s HSD test.

### 2.10. Repellence in Filter Paper Disc Bioassay

Repellent activity of hop extracts was further evaluated using the area preference method [24]. A filter paper disc (Whatman No. 1, Ø 8.0 cm, area = 50.2 cm^2^) was divided in half. One half was treated with 500 µL of plant extracts solution using a micropipette and the other half was treated with an equal volume of the respective solvent used as control. Both treated and control halves were air-dried for about 10 min to allow complete solvent evaporation, joined with transparent adhesive tape and the full disc fixed on the bottom of a Petri dish (Ø 9.0 cm). Ten weevil unsexed adults were confined to each filter paper disc within a metal O-ring (Ø 8.0 × 4.0 cm) covered with metallic net (mesh 1 mm) to prevent insect escape. The experiment was run in the dark at 26 ± 2 °C and 60 ± 5% r.h. Solutions of hop extracts, prepared as described in Section 2.4, were tested corresponding to the doses of 0.37, 0.75, 1.49, and 2.98 mg/cm^2^, respectively. Each bioassay was replicated 4 times. The number of weevils on the treated (Nt) and control (Nc) portion of paper disc was recorded at the following intervals: 10, 30 min, 1, 2, and 24 h, respectively. Percentage repellency (PR) values were calculated as follows:

PR = (Nc − Nt)/(Nc + Nt) × 100(6)

where positive PR values indicate repellence, whereas negative values indicate attraction. For each dose, PR values at different exposure times were submitted to ANOVA followed by Duncan’s HSD test for separation of means.

### 2.11. Electroantennography (EAG)

The olfactory sensitivity of male and female *S. granarius* antennae to ascending concentrations of the three hop extracts was assessed by EAG using the technique reported in previous studies [29,30]. Briefly, the head of a 2- to 3-week-old insect was excised from the prothorax using a scalpel and mounted between two glass capillary electrodes (Microglass, Naples, Italy) filled with Kaissling saline solution [31]. The recording electrode (diameter~100 μm) was put in contact with the dorsal surface of the antennal club while the neutral electrode was inserted into the base of the head. The electrical continuity between the antennal preparation and an AC/DC UN-6 amplifier in DC mode was maintained using AgCl-coated silver wires. The amplifier was connected to a PC equipped with the EAG 2.0 program (Syntech Laboratories, Hilversum, The Netherlands).

Five two-fold dilution of n-hexane, methanol, and acetone hop cone extracts (100, 50, 25, 12.5, 6.25 µg/µL) in the corresponding solvents (Sigma–Aldrich, Milan, Italy) were prepared. The test stimulus (10 µL of an extract solution) was adsorbed onto a filter paper (Whatman No. 1) strip (2 cm^2^) inserted in a Pasteur pipette (15 cm long) after solvent evaporation. The vapor stimuli (3 cm^3^) were puffed, using a disposable syringe, for 1 s into a charcoal-filtered and humidified air stream (500 mL/min) flowing over the antenna through a stainless-steel delivery tube (Ø 1.0 cm) with the outlet positioned~1 cm from the antenna. Control (10 μL of a solvent) and standard (10 μL of a 10 μg/μL (Z)-3-hexenol solution) stimuli were also applied at the beginning of the experiment and after each group of 5 test stimuli. The intervals between stimuli were 1 min. Each dose of the three hop extracts was tested on three antennae from different males and females.

EAG responses were measured by the maximum amplitude of negative polarity deflection (-mV) elicited by a stimulus [32]. To compensate for solvent and/or mechanosensory artefacts, the amplitude (mV) of the EAG responses to each test stimulus was subtracted by the mean EAG response of the two nearest solvent controls [33]. To compensate for the decrease in the antennal responsiveness during the experiment, the resulting EAG amplitude was corrected according to the reduction of the EAG response to the standard stimulus [34]. Dose–response curves were calculated based on the corrected EAG values. To verify antennal activation, the corrected mean male and female EAG response to the last dilution of each hop extract was compared to “0” value using one-sample Student’s *t*-test and regarded as “activated” if significant at *p* = 0.05. Saturation level was taken as the lowest dilution at which the mean response was equal to or less than the previous one [35]. Mean male and female EAG responses to each stimulus were compared using a Student’s *t*-test for independent samples at *p* = 0.05. Since no significant differences were found between the male and female EAG responses to each test stimulus, individual male and female EAG responses were pooled and analyzed together. For each dose tested, the mean EAG responses of adult insects to the three hop extracts were submitted to ANOVA followed by Tukey HSD test (*p* = 0.05). Levene’s test was used to assess homogeneity of variances.

## 3. Results

### 3.1. Contact and Inhalation Toxicity

Mortalities of *S. granarius* adults obtained 24 and 48 h after topical application of hop extracts are reported in Table 1, Table 2 and Table 3. For all samples, a dose-dependent increased mortality was found. Twenty-four hours after extract application, mortalities were significantly higher than the control starting from the 9.37 µg/adult dose of *n*-hexane extract (Table 1), and 18.75 µg/adult of both acetone (Table 2) and methanol (Table 3) extracts; 48 h after application, n-hexane and acetone extracts showed a decrease in the lowest active dose which was 4.69 and 9.37 µg/adult, respectively. Contact toxicity, 24 h after topical application returned LD_50_ values of 16.17, 25.77, and 31.07 µg/adult and LD_90_ values of 33.20, 42.64, and 49.48 µg/adult for acetone, *n*-hexane, and methanol extracts, respectively; these values slightly decreased 48 h after application (Table 1, Table 2 and Table 3).

Negligible mortality in inhalation toxicity assays was found for all the extracts in the dose range tested (0.018–1.111 g/L hop extract residue, data not shown).

### 3.2. Ingestion Toxicity, Antifeedant and Nutritional Activity

Ingestion toxicity and nutritional effects of hop extracts towards adult granary weevils are presented in Table 4, Table 5 and Table 6. Methanol (Table 4) and *n*-hexane extracts (Table 6) induced low insect mortality values only at the highest dose checked; a more toxic effect was shown by the acetone extract which caused about 60% insect mortality at the highest dose (Table 5). The highest dose of each extract significantly decreased the relative growth rate (RGR, F = 4.73–10.43; df = 5; *p* < 0.001–0.004), the efficiency conversion of ingested food (ECI, F = 4.58–11.14; df = 5; *p* < 0.001–0.005), and the relative consumption rate (RCR, F = 3.78–8.73; df = 5; *p* < 0.001–0.012), except for RCR in the case of acetone extract treatment. Doses of all extracts elicited positive FDI values; a significant dose-dependent increase in this value (FDI, F = 0.93–11.58; df = 4; *p* < 0.001–0.467) was found for methanol and *n*-hexane extracts, but not for the acetone one.

### 3.3. Anticholinesterase Activity

**Figure 1:**
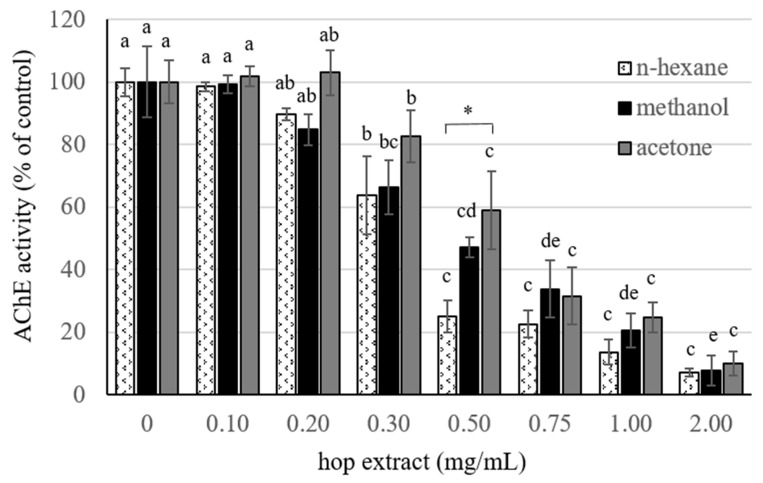
The anticholinesterase (AChE) activity of hop extracts. Mean values (±SE) of AChE activity obtained either in the absence or in the presence of different doses of hop *n*-hexane, methanol, and acetone extracts. Values (*n* = 3) were calculated as % of the control (enzyme activity measured in the absence of hop extracts). Among each series (hop extract), different letters indicate a significant difference (*p* < 0.05, Tukey’s HSD test); among each dose, * indicate a significant difference (*p* < 0.05, Tukey’s HSD test).

The effect of the different extracts on the AChE was investigated (Figure 1). For each hop extract, a dose-dependent inhibitory activity was found (*n*-hexane extract: F = 51.21, df = 7, *p* < 0.001; methanol extract: F = 34.41, df = 7, *p* < 0.001; acetone extract: F = 25.60, df = 7, *p* < 0.001). No significant differences among the samples were found, except for the dose of 0.50 μg/mL. Since in these experiments Tween-20 (0.4%, *v*/*v*) was used to resuspend *n*-hexane extract in phosphate buffer, the control was made so that the Tween-20 (up to 1%, *v*/*v*) did not affect enzyme activity. IC_50_ was calculated for each extract returning the following values: 0.331 ± 0.025, 0.440 ± 0.108, and 0.505 ± 0.041 μg/mL for *n*-hexane, methanol, and acetone extract, respectively.

### 3.4. Two-Choice Behavioural Bioassays

**Figure 2:**
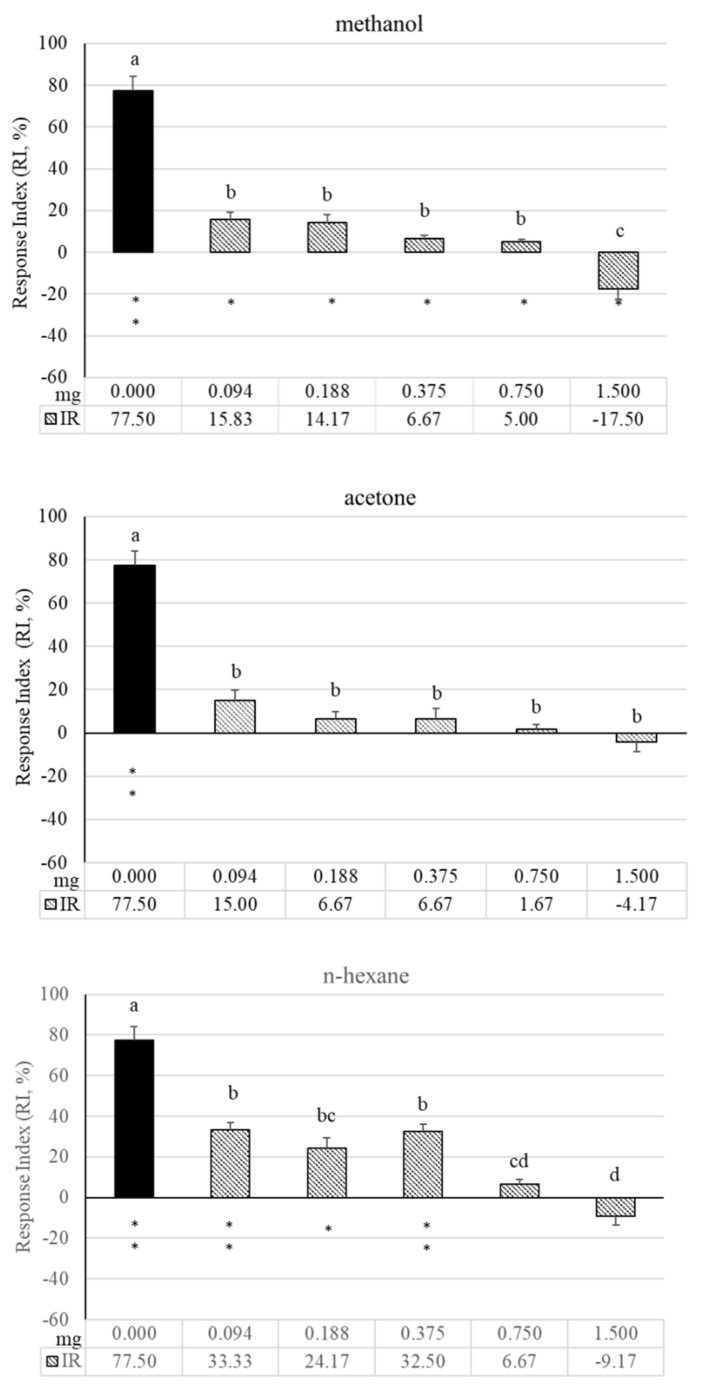
Response index (RI) of *S. granarius* adults to odors of wheat grains (200 g) alone (black bars) or in the presence of ascending doses of hop extracts in two-choice bioassays. For each set of experiments, values with the same letter are not significantly different (*p* < 0.05, Tukey’s HSD test); asterisks indicate significant differences between the number of insects in the treatment and the control (* *p* < 0.05, ** *p* < 0.01; Student’s *t*-test).

Results of two-choice behavioral bioassays evaluating the possible disruptive effects of different hop extracts on granary weevil orientation towards wheat grain odors are reported in Figure 2. Wheat grain odors elicited a highly positive and significant RI, indicating insect attraction. In the dose range tested, the RI to wheat grains was significantly decreased by the presence of methanol (F = 57.11, df = 5, *p* < 0.001), acetone (F = 41.19, df = 5, *p* < 0.001), and *n*-hexane extract (F = 40.46, df = 5, *p* < 0.001) with methanol and *n*-hexane extracts showing a dose-dependent effect. For these extracts, the highest dose (1.50 mg) resulted in negative and significant RIs, indicating actual repellency (Figure 2), with methanol extract eliciting the highest repellent effect (−17.50).

### 3.5. Repellence in Filter Paper Disc Bioassay

**Figure 3:**
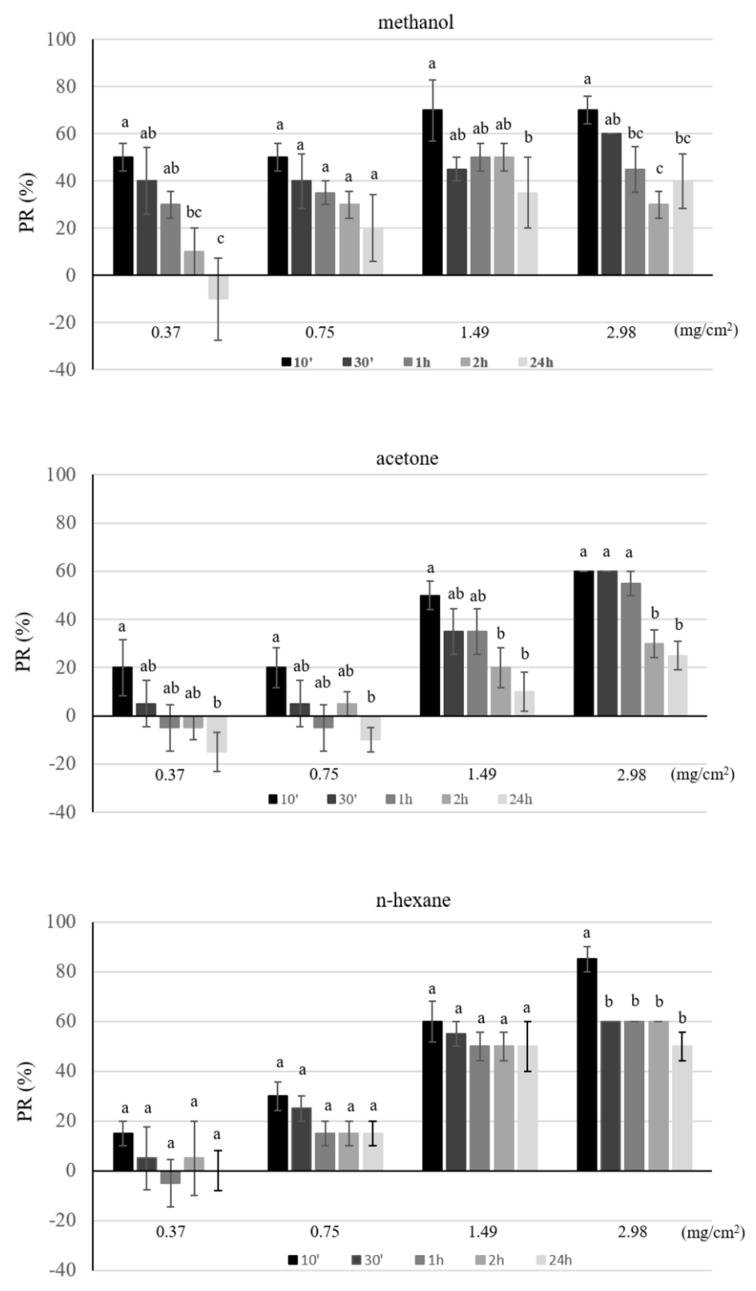
Percent repellency (PR) of different concentrations of hop extracts against *S. granarius* adults in filter paper disc bioassays. PR mean value (±S.E.), calculated as reported in the Methods section, obtained in four different experiments, were reported as a function of both dose and time after exposure. For each dose, different letters indicate significant differences among time (*p* < 0.05, Duncan MRT’s test).

Contact repellency of different hop extracts was further evaluated in filter paper disc bioassays (Figure 3). In the dose range tested, all the extracts repelled insects with the methanol one being the most active at the lowest dose tested (F = 5.61, df = 2, *p* < 0.05). Repellent activity was found to significantly decrease as a function of time of application, particularly for the acetone and methanol extract (methanol extract: F = 1.47–4.37, df = 4, *p* < 0.016–0.260; acetone extract: F = 1.77–17.55, df = 4, *p* < 0.001–0.188) (Figure 3). The two higher doses of the *n*-hexane extract exhibited a remarkable contact repellency over time.

### 3.6. EAG

**Figure 4:**
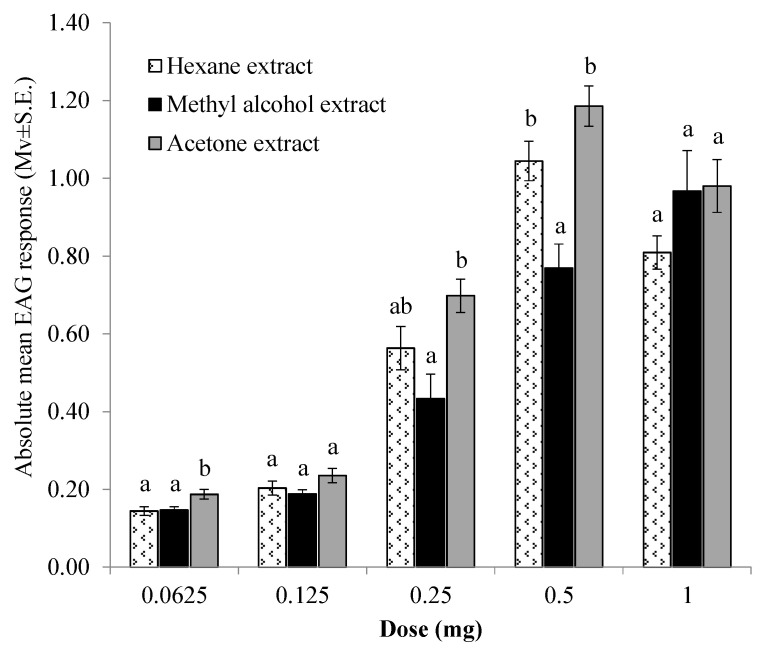
EAG responses (mean values ± SE) of adult *S. granarius* antennae (*n* = 6) to ascending doses of different hop cone extracts. For each dose, different letters indicate significant differences at (*p* < 0.05; Tukey’s HSD test).

The antennal sensitivity of granary weevil adults to increasing doses of *n*-hexane, methanol, and acetone hop cone extracts is presented in Figure 4. In the dose range tested, all extracts elicited measurable (*p* < 0.05 in all one-sample Student’s *t*-test) and dose-dependent EAG responses in both sexes without significant differences between males and females (*n*-hexane extract: *t* = 0.402–1.687, df = 4, *p* = 0.096–0.708; methanol extract: *t* = 0.340–1.478; df = 4, *p* = 0.214–0.751; acetone extract: *t* = 0.276–2.325; df = 4, *p* = 0.081–0.797). The amplitude of the mean EAG response to *n*-hexane and acetone decreased from the 0.5 to 1 mg doses, indicating saturation of receptors at the lowest one.

At the lowest dose tested, the mean EAG response to acetone extract was significantly higher than those to *n*-hexane and methanol extracts (F = 4.91, df = 2, *p* < 0.023). The mean EAG responses elicited by the same extract were significantly higher than those of methanol extract at the 0.25 (F = 5.85, df = 2, *p* = 0.013) and 0.5 mg (F = 14.83, df = 2, *p* < 0.001) doses, but not statistically different than those recorded at the same doses of the *n*-hexane extract.

## 4. Discussion

As part of the ongoing research on the biological activity of hop plant, results reported in this study shows that *n*-hexane, methanol, and acetone hop extracts exert different bioactivities against the pest *S. granarius*. Topical application of all three extracts resulted in a high contact mortality, reaching the 100% value at the highest dose tested (75.00 μg/adult). Among the extracts, the *n*-hexane was found to be the most active showing significant mortality starting from 9.37 µg/adult. The high contact toxicity of hop extracts was supported by the respective LD_50_ values which were comparable among them and lower than the values reported for similar extracts of other plants, such as *Scrophularia canina* L. [36] and *Dittrichia viscosa* (L.) [8], against the same insect species. However, LD_50_ value of acetone extract, which was found to be the most toxic, was about 4-fold higher than that of the pyrethrin extract (DL_50_ 4.29 µg/adult) against the congener *S. zeamais* Motschulsky [37]. Moreover, lower insecticidal activity for hexane and acetone root extracts of *Decalepis hamiltonii* against *S. oryzae* (L.) was found [38]. Interestingly, acetone and especially methanol extracts obtained from wild hop used in this study appeared to be more toxic than those obtained from a different hop ecotype against *S. granarius* [39], thus confirming the importance of ecotypes in determining hop properties [40,41,42]. A possible mechanism by which hop extracts exert contact toxicity may rely on the anticholinesterase action. In fact, all of them showed a dose-dependent inhibition of this enzyme, with limited differences in IC_50_ values among the extracts. In this regard, the absence of major differences in both LD_50_ and enzyme IC_50_ among the several extracts strongly support the hypothesis of anti-AChE mediated toxic activity. Notice that anticholinesterase activity was already found in water and ethanolic extracts of several hop ecotypes [43], but not in the EO of the same ecotype used in this study [21].

Hop extracts showed different ingestion toxicity. In particular, the acetone extract was the most active, reaching about 60% mortality at the highest dose, whereas the methanol was the less active, causing only 16% mortality at the same dose. These findings further suggest the occurrence of different active compounds in the extracts. Notice that no ingestion toxicity was reported for extracts of other plants, such as *S. canina* [36] and *D. viscosa* [8], against the same insect pest as well as for the plant-based commercial product Margosan^®^ (0.25% azadirachtin) [26].

In addition to ingestion toxicity, the highest doses of hop extracts affected nutritional indices with methanol extract showing the highest deterrence (about 75%) and the acetone extract providing the lowest ECI. The low conversion indices of ingested food may explain the higher mortality caused by ingestion observed for the acetone extract. Insect nutritional parameters obtained in the control (untreated disks flour) were in fairly good agreement with those calculated in previous studies [8,24,44]. Notice that the antifeedant activity of hop extracts is not unique since similar properties were reported for extracts of several plant species, particularly for methanolic extracts [45,46].

In distinction with what reported for similar plant extracts [38,47,48], hop extracts did not show any inhalation toxicity. A moderate inhalation toxicity (LC_50_ 136.37 mg/L; LC_90_ 201.48 mg/L) was found for the EO obtained from the same hop ecotype [21]. These differences between solvent extracts and EO could be due to the occurrence of different bioactive compounds or to the loss of the volatile compounds exerting inhalation toxicity during crude extracts preparation. In this regard, resins, essential oil, and polyphenols are the main components of hop cones [49]. Hop resin is usually classified as soft and hard: the former contains α- and β-acids (humulones and lupulones, respectively) and is characterized by a good solubility in *n*-hexane [50]; the latter, probably deriving from the oxidation of the soft resin, is completely insoluble in hexane, but soluble in methanol and diethyl ether [50]. Thus, we can speculate that hard resin was restricted to the methanol extract, whereas the n-hexane fraction was enriched in α- and β-acids. In addition to resin, further active components of hop cones are polyphenols, which include flavonols, flavan-3-ols, phenolic carboxylic acids, and others phenolic compounds as prenylflavonoids (xanthohumol, isoxanthohumol, desmethylxanthohumol, and 6- and 8-prenylnaringenin) [51]. For some of these, such as xanthohumol [39] and catechin [52], insecticidal activity was reported. However, identification of possible active components in these extracts strictly requires their chemical characterization since the relative abundance of compounds in hop cones is highly affected by both intrinsic and extrinsic factors, such as the variety and the agronomic-environmental conditions, respectively [53,54,55,56].

The observed repellency of hop extracts was investigated by using two different experimental approaches in order to assess both contact and short-range effects. All hop extracts exerted contact repellency towards granary weevil with the methanol extract being active even at low doses. Interestingly, the *n*-hexane extract maintained a good contact repellency over time with respect to other extracts, suggesting a possible long-lasting activity. The disruptive effects of hop extracts on adult granary weevil orientation were also exerted at a distance and even in the presence of a highly attractive food source, as shown by the arena behavioral bioassays with wheat grains. In these experiments, the attractiveness of wheat grains was significantly reduced by the presence of all the extracts, with the methanol one exerting actual repellency at the highest dose. In agreement with these results, electrophysiological tests confirmed the presence in all the extracts of volatile compounds able to stimulate the peripheral olfactory system of granary weevil males and females in a dose-dependent manner. Repellent extracts may be used to control hidden infestation before fresh grain is introduced [57] or they may be incorporated into packaging materials to prevent insects from entering packaged cereal [58].

All these findings strongly suggest good potential of hop crude extracts for the development of sustainable control strategy of this pest.

## 5. Conclusions

In this study, we showed that methanol, acetone, and *n*-hexane extracts of hop exerted a significant bioactivity against *S. granarius* adults. All the extracts showed contact and short-range repellent effects and, more importantly, reduced the attractiveness of stored food. In particular, a good contact and a moderate ingestion toxicity was found for the acetone extract. Although differences in bioactivity among the different extracts were found, all of them provided interesting results and are worthy of further investigation in order to identify the bioactive compounds.

## Figures and Tables

**Table 1:** Contact toxicity of different concentrations of *n*-hexane extract against *S. granarius* adults 24 and 48 h after topical application. For each exposure time, mean mortality values followed by same letter are not significantly different at *p* ≤ 0.05 (Tukey HSD test).

Dose (µg/Adult)	Exposure Time (h)	% Mortality (Mean ± S.E.)	Regression Equation	χ^2^	LD_50_ (95% CL, µg/Adult)	LD_90_ (95% CL, µg/Adult)
75.00	24 h	100.00 ± 0.00 a	*y* = 3.33x − 4.24	8.77	25.77 (20.34–34.50)	42.64 (34.05–61.18)
37.50		77.50 ± 4.50 b				
18.75		32.50 ± 7.50 c				
9.37		22.50 ± 5.90 c				
4.69		2.50 ± 2.50 d				
2.34		0.00 ± 0.00 d				
0.00		0.00 ± 0.00 d				
F		95.88				
d.f.		6				
*p*		<0.001				
75.00	48 h	100.00 ± 0.00 a	*y* = 0.087x − 2.027	10.60	22.94 (17.79–31.08)	38.69 (30.67–56.18)
37.50		85.00 ± 3.27 a				
18.75		37.50 ± 4.53 b				
9.37		27.50 ± 6.50 b				
4.69		5.00 ± 3.27 c				
2.34		0.00 ± 0.00 d				
0.00		0.00 ± 0.00 d				
F		128.68				
d.f.		6				
*p*		<0.001				

**Table 2:** Contact toxicity of different concentrations of acetone extract against *S. granarius* adults 24 and 48 h after topical application. For each exposure time, mean mortality values followed by same letter are not significantly different at *p* ≤ 0.05 (Tukey HSD test).

Dose (µg/Adult)	Exposure Time (h)	% Mortality (Mean ± S.E.)	Regression Equation	χ^2^	LD_50_ (95% CL, µg/Adult)	LD_90_ (95% CL, µg/Adult)
75.00	24 h	100.00 ± 0.00 a	*y* = 4.10x − 4.38	16.37	16.17 (9.65–28.85)	33.20 (20.96–157.85)
37.50		97.50 ± 2.50 a				
18.75		57.50 ± 4.53 b				
9.37		7.50 ± 3.66 c				
4.69		5.00 ± 3.27 c				
2.34		0.00 ± 0.00 c				
0.00		0.00 ± 0.00 c				
F		290.48				
d.f.		6				
*p*		<0.001				
75.00	48 h	100.00 ± 0.00 a	*y* = 3.84x − 4.51	6.21	14.91 (12.82–17.41)	32.14 (26.29–42.77)
37.50		97.50 ± 2.50 a				
18.75		60.00 ± 5.34 b				
9.37		15.00 ± 3.27 c				
4.69		7.50 ± 3.66 cd				
2.34		0.00 ± 0.00 d				
0.00		0.00 ± 0.00 d				
F		241.28				
d.f.		6				
*p*		<0.001				

**Table 3:** Contact toxicity of different concentrations of methanol extract against *S. granarius* adults 24 and 48 h after topical application. For each exposure time, mean mortality values followed by same letter are not significantly different at *p* ≤ 0.05 (Tukey HSD test).

Dose (µg/Adult)	Exposure Time (h)	% Mortality (Mean ± S.E.)	Regression Equation	χ^2^	LD_50_ (95% CL, µg/Adult)	LD_90_ (95% CL, µg/Adult)
75.00	24 h	100.00 ± 0.00 a	*y* = 0.07x − 2.163	2.25	31.07 (27.33–36.03)	49.48 (43.19–59.09)
37.50		67.50 ± 5.26 b				
18.75		17.50 ± 4.53 c				
9.37		7.50 ± 3.66 cd				
4.69		5.00 ± 3.27 cd				
2.34		2.50 ± 2.50 d				
0.00		0.00 ± 0.00 d				
F		137.14				
d.f.		6				
*p*		<0.001				
75.00	48 h	100.00 ± 0.00 a	*y* = 0.06x − 1.89	0.64	28.66 (25.01–33.52)	48.08 (41.71–57.81)
37.50		72.50 ± 3.66 b				
18.75		22.50 ± 5.90 c				
9.37		12.50 ± 3.66 cd				
4.69		5.50 ± 3.27 d				
2.34		5.50 ± 3.27 d				
0.00		2.50 ± 2.50 d				
F		118.39				
d.f.		6				
*p*		<0.001				

**Table 4:** Nutritional indices, mortality, and food deterrence of *S. granarius* adults of different concentrations of methanol extract. Means in the same column with the same letter are not significantly different at the 0.05 level determined by the Tukey’s HSD test.

Concentration (µg/Disk)	Mortality (%)	RGR ^1^	RCR	ECI	FDI (%)
750.00	16.00 a	−0.011 ± 0.008 a	0.066 ± 0.047 a	−31.915 ± 11.695 a	74.000 ± 20.199 a
375.00	4.00 b	−0.020 ± 0.006 ab	0.188 ± 0.009 b	−10.363 ± 2.465 b	27.951 ± 7.782 b
187.50	0.00 b	−0.0130 ± 0.007 b	0.199 ± 0.007 b	−6.304 ± 2.620 b	21.222 ± 7.185 b
93.75	0.00 b	−0.003 ± 0.003 c	0.230 ± 0.015 b	−1.134 ± 1.173 b	12.621 ± 4.576 b
46.87	0.00 b	−0.001 ± 0.002 c	0.224 ± 0.018 b	−0.416 ± 0.780 b	15.780 ± 4.405 b
Control	0.00 b	0.012 ± 0.003 c	0.278 ± 0.039 b	6.973 ± 0.513 b	

^1^ RGR, relative growth rate; RCR, relative consumption rate; ECI, efficiency conversion of ingested food; FDI, feeding deterrent index.

**Table 5:** Nutritional indices, mortality, and food deterrence of *S. granarius* adults of different concentrations of acetone extract. Means in the same column with the same letter are not significantly different at the 0.05 level determined by the Tukey’s HSD test.

Concentration (µg/Disk)	Mortality (%)	RGR ^1^	RCR	ECI	FDI (%)
750.00	62.00 a	−0.091 ± 0.020 a	0.122 ± 0.024 ab	−86.710 ± 20.378 a	41.033 ± 13.712 a
375.00	34.00 ab	−0.035 ± 0.009 ab	0.110 ± 0.020 a	−46.266 ± 21.573 abc	55.427 ± 9.801 a
187.50	40.00 ab	−0.060 ± 0.015 a	0.129 ± 0.043 ab	−75.607 ± 26.856 ab	44.223 ± 21.273 a
93.75	16.00 bc	−0.048 ± 0.016 ab	0.123 ± 0.036 ab	−65.118 ± 24.932 abc	48.037 ± 16.664 a
46.87	4.00 c	0.008 ± 0.007 bc	0.207 ± 0.016 ab	3.228 ± 3.222 bc	18.489 ± 5.192 a
Control	0.00 c	0.023 ± 0.077 c	0.242 ± 0.020 b	9.028 ± 2.166 c	

^1^ RGR, relative growth rate; RCR, relative consumption rate; ECI, efficiency conversion of ingested food; FDI, feeding deterrent index.

**Table 6:** Nutritional indices, mortality, and food deterrence of *S. granarius* adults of different concentrations of *n*-hexane extract. Means in the same column with the same letter are not significantly different at the 0.05 level determined by the Tukey’s HSD test.

Concentration (µg/Disk)	Mortality (%)	RGR ^1^	RCR	ECI	FDI (%)
750.00	38.00 a	−0.015 ± 0.002 a	0.132 ± 0.009 a	−11.596 ± 1.410 a	40.463 ± 6.509 a
375.00	10.00 b	−0.008 ± 0.004 a	0.161 ± 0.003 ab	−5.248 ± 2.352 ab	21.816 ± 2.537 ab
187.50	6.00 b	−0.003 ± 0.011 ab	0.186 ± 0.016 bc	−0.529 ± 6.876 ab	4.827 ± 5.844 b
93.75	0.00 b	0.006 ± 0.002 ab	0.189 ± 0.009 bc	3.337 ± 1.064 b	4.616 ± 3.320 b
46.87	0.00 b	0.005 ± 0.003 ab	0.190 ± 0.007 bc	2.756 ± 1.426 b	7.376 ± 3.119 b
Control	0.00 b	0.016 ± 0.002 b	0.204 ± 0.009 c	7.735 ± 0.854 b	

^1^ RGR, relative growth rate; RCR, relative consumption rate; ECI, efficiency conversion of ingested food; FDI, feeding deterrent index.

## Data Availability

The datasets generated during and/or analyzed during the current study are available from the corresponding authors on reasonable request.
